# The Impact of the Initial Clinical Presentation of Bladder Cancer on Histopathological and Morphological Tumor Characteristics

**DOI:** 10.3390/jcm12134259

**Published:** 2023-06-25

**Authors:** Dora Jakus, Ivana Šolić, Ivan Jurić, Josip A. Borovac, Marijan Šitum

**Affiliations:** 1 Department of Urology, University Hospital Center Split, 21000 Split, Croatia; ivana.solic1@gmail.com (I.Š.); ivan.juric201@gmail.com (I.J.); msitum@kbsplit.hr (M.Š.); 2 Clinic for Heart and Vascular Diseases, University Hospital Center Split, 21000 Split, Croatia; josip.borovac@me.com

**Keywords:** urinary bladder neoplasms, hematuria, lower urinary tract symptoms, asymptomatic diseases, early detection of cancer

## Abstract

This study investigated the impact of the initial clinical presentation of bladder cancer on tumor characteristics. A cross-sectional, retrospective study was performed, and it involved 515 patients who underwent transurethral bladder cancer resection at the University Hospital Center Split between April 2019 and April 2023, excluding recurrent cases. The association between symptomatic versus asymptomatic presentation and bladder cancer characteristics was analyzed. A subgroup analysis compared tumor characteristics between patients with gross and microscopic hematuria. Multiple regression analyses revealed a significant association between symptomatic presentation and the detection of high-grade bladder cancer (OR 3.43, 95% CI 2.22–5.29, *p* < 0.001), concomitant CIS (OR 3.41, 95% CI 1.31–8.88, *p* = 0.012), T2 stage bladder cancer (OR 5.79, 95% CI 2.45–13.71, *p* < 0.001), a higher number of tumors (IRR 1.24, 95% CI 1.07–1.45, *p* = 0.005), and larger tumor size (B 1.68, 95% CI 1.19–2.18, *p* < 0.001). In the subgroup analysis, gross hematuria was associated with the detection of high-grade bladder cancer (OR 2.07, 95% CI 1.12–3.84, *p* = 0.020), T2 stage bladder cancer (OR 6.03, 95% CI 1.42–25.49, *p* = 0.015), and larger tumor size (B 1.8, 95% CI 0.99–2.6, *p* < 0.001). The identified associations between symptomatic presentation and unfavorable bladder cancer characteristics, likely attributed to early detection in asymptomatic cases, underscore the importance of additional research in the development of bladder cancer screening strategies.

## 1. Introduction

According to GLOBOCAN 2020, bladder cancer was the 10th most prevalent malignancy in the world [1]. Bladder cancer is the most prevalent malignant disease of the urinary tract [2]. Approximately 80% of patients experience hematuria, which is the predominant presenting symptom. Hematuria is typically intermittent, gross, and painless, but it may also present as microscopic hematuria [3,4]. Roughly 20% of patients present with irritative voiding symptoms, such as dysuria, urgency, frequency, or urge incontinence. These symptoms are usually associated with invasive bladder cancer or carcinoma in situ (CIS). Less commonly, initial symptoms include obstructive urinary disorders. Patients with advanced stages of bladder cancer may initially present with abdominal pain, lower extremity edema due to the compression of iliac vessels, or flank pain resulting from the obstruction of the ureters [5].

Due to an increase in the use of laboratory procedures and imaging methods in daily clinical practice, bladder cancer diagnosis in patients without any symptoms is becoming more frequent. Some studies have reported that the majority of incidentally detected bladder cancers diagnosed by imaging methods were low-stage and low-grade [6,7]. Moreover, the presence of gross hematuria was observed to have a correlation with a higher stage of disease at the time of diagnosis, as opposed to microscopic hematuria [8]. As a large percentage of non-muscle invasive bladder cancers are related to a good prognosis, early diagnosis is of the essence [9]. Compared to other malignancies, such as kidney tumors, the diagnosis of bladder cancer in asymptomatic patients has been poorly studied, with insufficient information on cancer characteristics and prognosis [10].

Our study aims to compare the histopathological and morphological characteristics of bladder cancer between patients who presented with symptoms and asymptomatic patients (either incidentally diagnosed during imaging studies or diagnosed during the evaluation of asymptomatic microscopic hematuria). We hypothesized that asymptomatic patients would exhibit more favorable histopathological and morphological bladder cancer features due to early detection, namely, prior to the manifestation of symptoms.

## 2. Materials and Methods

### 2.1. Study Design and Participants

We conducted a cross-sectional, retrospective study at a single center. The participants included in the study were adult patients who underwent transurethral resection of primary bladder tumor (TURBT) at the University Hospital Center Split between April 2019 and April 2023. Only patients with a confirmed diagnosis of urothelial bladder carcinoma upon final histopathological analysis were enrolled, whereas those with benign lesions or non-urothelial malignancies were excluded from the study. Patients with recurrent tumors were also excluded, and so were those with incomplete medical records.

### 2.2. Data Collection and Definition of Variables

The data were retrieved from the electronic medical records of our institution. Patients were assigned identification numbers with the aim of maintaining their anonymity throughout the study. The collected data included patient demographics, the mode of initial clinical presentation, and bladder cancer characteristics.

Demographic data of interest were the patients’ sex, age, smoking history, comorbidities, and past medical history.

The mode of initial clinical presentation was classified as either symptomatic or asymptomatic. Patients who presented with gross hematuria (the presence of blood in urine observed by the patient and/or urologist), voiding symptoms (irritative or obstructive), and abdominal pain were categorized as symptomatic. Patients who were defined as asymptomatic did not present with any of the symptoms related to bladder cancer. The detection of cancer in these patients was either incidental (during imaging studies performed for unrelated reasons) or prompted by the presence of microscopic hematuria without any other accompanying symptoms. Microscopic hematuria was determined by the presence of three or more erythrocytes per high-power microscopic field in the urine sediment [11].

The collected data also included histopathological and morphological bladder cancer features. The considered histopathological characteristics were the stage and grade of bladder cancer, determined by a pathological examination of the specimen after primary TURBT and second-look resection (when indicated) (Figure S1). These characteristics were defined following the classification of the World Health Organization and the TNM Classification for Bladder Cancer [12,13]. The morphological characteristics of interest were the number of tumors and total tumor size. These data were extracted from written descriptions of the surgical procedures (TURBT) provided by the operating urologists. All operating urologists followed a consistent approach when documenting the number and size of tumors in their surgical reports, in accordance with the agreements and standards established by our institution. For each patient, the number of tumors was described as one if the urologist documented and resected a single tumor, or more if the findings indicated the presence of multiple tumors. The size assessment was based on the largest dimension of the tumor, as determined by the operating urologist, namely, using the cutting loop of the resectoscope (measuring 6 mm) as a reference (Figure S2). In cases where patients had multiple tumors, the total tumor size was calculated by summing the largest dimensions of all the tumors.

### 2.3. Outcomes

We investigated the association between the following:

1. Demographic characteristics of patients;
2. The mode of initial clinical presentation (symptomatic vs. asymptomatic);

and the following bladder cancer features:

1. Histopathological characteristics;
2. Morphological characteristics.

Subsequently, we separated the subgroup of patients with hematuria from the study population and compared the aforementioned bladder cancer characteristics between patients with gross hematuria and microscopic hematuria.

### 2.4. Statistical Analysis

The Kolmogorov–Smirnov test was utilized to assess the normality of data distribution. Categorical variables are displayed as numbers (n) and corresponding percentages (%). The Chi-square test was applied to compare distributions of categorical data. The median and interquartile ranges (IQR) were used to present the values of continuous variables. The Mann–Whitney test was employed to compare the differences in continuous data. To investigate the relationship between independent variables (demographic data of patients and the mode of initial clinical presentation) and dependent variables of interest (bladder cancer characteristics), separate multiple logistic regression models were constructed for each dependent variable of interest. Multiple logistic regression was used for dichotomous dependent variables, while multiple Poisson regression and multiple linear regression were utilized for count and continuous dependent variables, respectively. All reported *p*-values were two-tailed, and a *p* < 0.005 was considered to be statistically significant. The IBM SPSS Statistics for Windows, Version 26.0 (IBM Corp., Armonk, NY, USA) software package was used to conduct all statistical analyses. 

## 3. Results

### 3.1. Baseline Patient Demographics and the Mode of Initial Clinical Presentation

A total of 515 patients with primary bladder cancer met the inclusion criteria. Among them, 71.8% of patients were male. The median age was 70 years (IQR 63–78 years), 59.6% of patients were current or former smokers, and the median number of associated comorbidities was 2 (IQR 1–3). 

**Figure 1:**
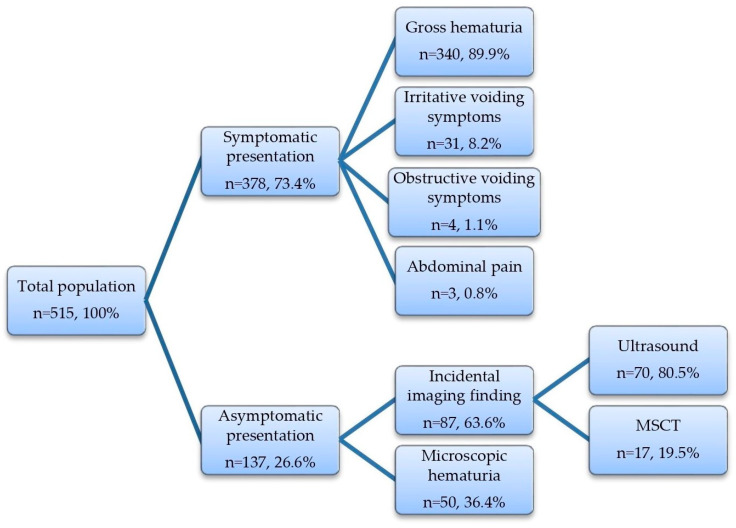
Mode of initial clinical presentation of bladder cancer in the studied population.

Figure 1 illustrates the distribution of patients according to the mode of initial presentation. For the majority of patients (n = 378, 73.4%), the diagnostic process was initiated due to the presence of bladder-cancer-related symptoms, primarily gross hematuria (89.9% of symptomatic patients). A subset of cases involved patients without any symptoms (n = 137, 26.6%), where bladder cancer was incidentally detected through imaging studies (in 63.6% of cases), with ultrasound being the most common modality (80.5% of incidental findings). In 36.4% of asymptomatic patients, the diagnostic process was prompted by the presence of microscopic hematuria.

### 3.2. Symptomatic vs. Asymptomatic Presentation

A comparison of baseline demographic data between symptomatic and asymptomatic patient groups is presented in Table 1. Apart from a slightly older age of symptomatic patients, no other significant differences in patient demographics were observed between the two groups. 

Table 1 also presents a comparison of histopathological and morphological bladder cancer characteristics between symptomatic and asymptomatic patients. The results showed a significantly lower percentage of PUNLMP/low-grade bladder cancer and a higher percentage of high-grade bladder cancer (*p* < 0.001) and concomitant CIS (*p* = 0.011) in the symptomatic group. Symptomatic patients were found to have a significantly lower frequency of Ta and a higher frequency of T1 and T2 bladder cancer stages (*p* < 0.001). The symptomatic group also exhibited a greater tumor burden in terms of both quantity (*p* = 0.002) and size (*p* < 0.001).

Multiple regression analyses (Table 2) revealed a significant association between symptomatic presentation and the following:

1. Detection of high-grade bladder cancer vs. PUNLMP/low-grade bladder cancer (*p* < 0.001);
2. Presence of concomitant CIS vs. no CIS (*p* = 0.012);
3. Detection of T2 stage bladder cancer vs. Ta/T1 stage bladder cancer (*p* < 0.001);
4. Detection of a higher number of tumors (*p* = 0.005);
5. Larger tumor size (*p* < 0.001).

Furthermore, there was a small but significant association between the age of patients and the presence of high-grade bladder cancer (*p* < 0.001) as well as an increased tumor number (*p* = 0.005) and size (*p* < 0.001). Male sex was also significantly associated with the presence of high-grade bladder cancer (*p* < 0.001).

### 3.3. Gross vs. Microscopic Hematuria

The subgroup of patients with hematuria, either gross or microscopic, was analyzed separately. Histopathological and morphological bladder cancer characteristics were compared between patients presenting with gross and microscopic hematuria (Table 3). The results showed a significantly lower percentage of PUNLMP/low-grade bladder cancer and a higher percentage of high-grade bladder cancer (*p* = 0.034) in the gross hematuria group. Patients with gross hematuria had a significantly lower frequency of Ta and a higher frequency of T1 and T2 bladder cancer stages (*p* = 0.002). Additionally, they had a larger median tumor size (*p* < 0.001). No differences were found between the two groups regarding the frequency of concomitant CIS and the number of tumors.

Multiple regression analyses (Table 4) demonstrated significant associations between gross hematuria and the following:

1. Detection of high-grade bladder cancer vs. PUNLMP/low-grade bladder cancer (*p* = 0.020);
2. Detection of T2 stage bladder cancer vs. Ta/T1 stage bladder cancer (*p* = 0.015);
3. Larger tumor size (*p* < 0.001).

Moreover, the age of patients exhibited a small but significant association with the presence of high-grade bladder cancer (*p* = 0.003) and an increased tumor number (*p* < 0.001) and size (*p* < 0.001). The male sex showed a significant association with the presence of high-grade bladder cancer (*p* = 0.032).

## 4. Discussion

Our study revealed significant associations between the mode of initial presentation and both histopathological and morphological bladder cancer characteristics. More than 70% of cancers in asymptomatic patients were PUNLMP/low-grade and Ta stage, pointing to less aggressive forms. These findings are in line with previous studies, which have shown that incidentally discovered bladder cancers during diagnostic imaging were primarily low-grade and Ta stage [6,7,14]. In contrast, symptomatic patients were diagnosed with high-grade cancers and cancers invading the lamina propria or detrusor muscle in more than 50% of cases, suggesting less favorable prognostic features.

Two recent retrospective studies have investigated incidentally diagnosed bladder cancer and compared the pathological features of incidental cases detected through imaging techniques with symptomatic cases. In both studies, the criteria for defining study groups slightly differed from ours, as they included patients with microscopic hematuria in the symptomatic group [14,15]. Since microscopic hematuria without additional symptoms is a sign of a potential disease observed by a physician, rather than a symptom, we classified patients with microscopic hematuria into the asymptomatic group [11]. Gaya et al. observed that incidentally diagnosed patients had smaller tumor sizes and lower frequencies of muscle-invasive and grade 3 bladder cancer compared to symptomatic cases. Consistent with their findings, our results demonstrated significantly lower frequencies of high-grade and higher-stage bladder cancer, as well as smaller tumor sizes in the asymptomatic group. In contrast, our investigation also included multiple regression analyses to examine the relationship between symptomatic presentation and unfavorable bladder cancer features more comprehensively, taking into account the influence of demographic predictors [14]. Kamecki et al. reported that incidental diagnosis was associated with a significantly lower likelihood of having high-grade (OR 0.52) and muscle-invasive or metastatic bladder cancer (OR 0.19) [15]. Similarly, our results showed that symptomatic patients had nearly 3.5 times higher odds of being diagnosed with high-grade bladder cancer and almost 6 times higher odds of having T2 stage bladder cancer. To the best of our knowledge, our study is the first to analyze and identify the association between the mode of initial clinical presentation and additional features of bladder cancer, alongside the previously studied grade and stage. In the symptomatic group, we observed a significantly higher frequency of concomitant CIS, a greater median number of tumors, and a larger median tumor size compared to the asymptomatic group. Moreover, our multiple regression analyses found a relationship between symptomatic presentation and the aforementioned additional unfavorable features of bladder cancer, including nearly 3.5 times higher odds of having concomitant CIS, a 24% increase in the number of tumors, and a tumor size that was 1.68 cm larger on average. These novel findings contribute to a deeper understanding of the relationship between the mode of initial clinical presentation and bladder cancer characteristics, enhancing our knowledge in this area.

Hematuria is the predominant and most closely linked symptom of bladder cancer [3,4,5]. Among the participants in our study, 340 presented with gross hematuria, while 50 were diagnosed based on the evaluation of microscopic hematuria in the absence of other accompanying symptoms. Although current guidelines recommend further evaluation of both gross and microscopic hematuria, there is evidence indicating that a considerable number of patients with microscopic hematuria are not referred for further urological assessment [11,16]. Given that a significant percentage of our study population experienced some form of hematuria (75.7%), and taking into account the tendency of healthcare providers to overlook microscopic hematuria, we isolated the subgroup of patients with hematuria and compared bladder cancer characteristics between those with gross and microscopic hematuria. Our results demonstrated an association between gross hematuria and unfavorable bladder cancer features. Patients with gross hematuria had approximately twice the odds of being diagnosed with high-grade bladder cancer, approximately 6 times higher odds of having T2 stage bladder cancer, and a tumor size that was 1.8 cm larger on average. The relationship between the severity of hematuria and bladder tumor characteristics has been poorly investigated to date. A study conducted by Ramirez et al. focused primarily on examining the impact of microscopic versus gross hematuria on bladder cancer grade and stage, without investigating additional tumor features. Their findings pointed to an association between gross hematuria and an elevated risk of invasive bladder cancer (OR 1.688 for T2 stage, OR 1.73 for T1 stage), while the risk of high-grade disease did not demonstrate a significant increase. It was not specified whether patients with microscopic hematuria were necessarily asymptomatic or if they may have experienced other bladder cancer-related symptoms, which may explain the substantial differences in risks compared to our results [8]. Further studies are needed to clarify these discrepancies.

In both the overall study population and the hematuria subgroup, we identified a modest, yet significant relationship between advanced age and adverse bladder cancer characteristics, which is in line with previous literature findings. Furthermore, we observed an association between male sex and high-grade bladder cancer, although conflicting evidence exists regarding this relationship in the literature [17,18]. 

Our findings can be attributed to the detection of bladder cancer in earlier development phases in asymptomatic patients, namely when tumors exhibit more favorable histopathological and morphological features. The progressive nature of bladder cancer is well-established and driven by the accumulation of genetic mutations in bladder cancer tissue over time. These genetic alterations contribute to the advancement of tumor grade and stage, as well as to an increase in tumor size and number [19,20]. Although our study did not investigate the associations between the mode of initial presentation and the prognosis of bladder cancer, the impact of unfavorable bladder cancer characteristics on poor outcomes is widely acknowledged. Previous studies have consistently identified the number of tumors, tumor size, T stage, grade, and the presence of concomitant CIS at the time of diagnosis as prognostic factors for bladder cancer recurrence and progression [21,22,23]. Moreover, previous research has demonstrated the significance of unfavorable histopathological and morphological characteristics in predicting metastasis development and influencing cancer-specific and overall survival rates [24,25,26,27]. The primary treatment for T2 bladder cancer, involving cystectomy and chemotherapy, is associated with a higher morbidity rate compared to the TURBT [28]. Consequently, early detection may play a significant role in potentially improving clinical outcomes and enhancing survival rates. A recently published study demonstrated a trend towards improved survival in patients incidentally diagnosed with bladder cancer compared to non-incidentally diagnosed cases [29].

In our study, we observed a significant proportion of bladder cancer cases (26.6%) being diagnosed in asymptomatic patients. The upsurge in the availability and usage of laboratory procedures and imaging methods in daily clinical practice may explain this phenomenon [30]. It is noteworthy that the majority of asymptomatic cases in our study were detected through ultrasound, a harmless and non-invasive method widely accessible in urological practice [31]. Another frequent cause of diagnosis in asymptomatic patients was the evaluation of microscopic hematuria detected in a simple urine sediment test. However, it is important to highlight that more than 70% of bladder cancer cases in our study population were detected only after the onset of symptoms, and these cases exhibited less favorable tumor features, potentially indicating delayed diagnoses. While routine bladder cancer screening in asymptomatic patients is currently not recommended due to a lack of evidence regarding its effectiveness, our findings may prove useful in directing additional future research on screening strategies, particularly in high-risk patients [32,33]. Special attention should be directed towards exploring the potential role of ultrasound and urine sediment analysis in bladder cancer screening, as both methods are simple, readily available, and non-invasive. Although there are limitations to the currently available screening methods, our findings support the idea that early diagnosis may still hold value in improving outcomes for patients with bladder cancer. 

Several limitations of our study should be acknowledged. Firstly, this was an observational, retrospective study. As such, it may have introduced biases and limitations in terms of data quality. Another limitation is that the study was conducted at a single center, which may impact the generalizability of the findings to a broader population. Additionally, the determination of the number and size of tumors relied on subjective assessments made by the operating urologists during TURBT, which may have introduced a potential limitation in terms of objectivity when measuring and reporting the said variables. Another limitation is the fact that we did not categorize microscopic hematuria based on the quantity of red blood cells, which limited our ability to perform sub-analyses and evaluate the impact of the severity of microscopic hematuria on bladder cancer characteristics. 

## 5. Conclusions

An increase in the use of laboratory procedures and imaging techniques has led to a rise in the detection of bladder cancer among asymptomatic patients. Our study revealed that asymptomatic patients who were incidentally diagnosed through imaging studies or identified during the evaluation of asymptomatic microscopic hematuria exhibited more favorable histopathological and morphological bladder cancer features compared to symptomatic patients. While previous studies have established an association between symptomatic presentation and a more advanced bladder cancer grade and stage, our study adds novel insights by identifying additional factors of interest, including the presence of concomitant CIS, larger tumor size, and increased number of tumors [14,15]. These findings may be attributed to the early detection of bladder cancer in asymptomatic patients. Further research is warranted to fully understand the implications of early detection and its long-term impact on patient outcomes.

## Figures and Tables

**Table 1:** Comparison of baseline patient demographics and bladder cancer features between symptomatic and asymptomatic patients.

Variable	Symptomatic Presentation		*p*-Value
	YES	NO	
Baseline demographic data			
Number of patients, n (%)	378 (73.4)	137 (26.6)	
Male	265 (70.1)	105 (76.7)	
Female	113 (29.9)	32 (23.3)	0.145 ^1^
Age (years), median (IQR)	71 (63–79)	69 (62–75)	0.043 ^2^
Smoking history, n (%)	229 (60.6)	78 (56.9)	0.456 ^1^
Number of comorbidities, median (IQR)	2 (1–3)	1 (0–3)	0.401 ^2^
Bladder cancer characteristics			
Bladder cancer grade, n (%)			
PUNLMP/low grade	157 (41.5)	97 (70.8)	
High-grade	221 (58.5)	40 (29.2)	<0.001 ^1^
Concomitant CIS, n (%)	41 (10.8)	5 (3.6)	0.011 ^1^
Bladder cancer stage, n (%)			
Ta	180 (47.6)	110 (80.3)	
T1	118 (31.2)	21 (15.3)	
T2	80 (21.2)	6 (4.4)	<0.001 ^1^
Morphological characteristics, median (IQR)			
Number of tumors	1 (1–3)	1 (1–2)	0.002 ^2^
Total tumor size (cm)	3.5 (2.2–5)	2 (1.15–3)	<0.001 ^2^

^1^ Chi-square test; ^2^ Mann–Whitney test. Abbreviations: CIS—carcinoma in situ; IQR—interquartile range; PUNPLMP—papillary urothelial neoplasm of low malignant potential.

**Table 2:** Results of multiple regression analyses examining the impact of patient demographics and symptomatic presentation on the histopathological grade, stage, number, and size of bladder cancer in the studied population.

	Dependent Variables				
Independent Variables	High Grade vs. Non-High-Grade	Concomitant CIS vs. no CIS	T2 Stage vs. Ta/T1 Stage	Number of Tumors	Tumor Size
	OR (95% CI), *p* Value ^1^	OR (95% CI), *p* Value ^1^	OR (95% CI), *p* Value ^1^	IRR (95% CI), *p* Value ^2^	B Coefficient (95% CI), *p* Value ^3^
Sex	1.62 (1.07–2.44), 0.022	2.12 (0.95–4.69), 0.065	0.68 (0.41–1.12), 0.129	0.99 (0.86–1.13), 0.857	−0.24 (−0.72–0.25), 0.332
Male					
Female (reference)					
Age	1.04 (1.02–1.06), <0.001	1 (0.97–1.04), 0.891	1.01 (0.98–1.04), 0.524	1.01 (1.006–1.02), <0.001	0.05 (0.03–0.07), <0.001
Smoking history	0.87 (0.59–1.28), 0.470	0.91 (0.47–1.76), 0.778	0.63 (0.38–1.05), 0.076	0.96 (0.85–1.09), 0.571	−0.32 (−0.78–0.14), 0.175
Yes					
No (reference)					
Number of comorbidities	0.91 (0.79–1.05), 0.186	0.88 (0.69–1.11), 0.282	0.96 (0.8–1.15), 0.668	0.99 (0.94–1.03), 0.542	−0.07 (−0.23–0.09), 0.400
Symptomatic presentation	3.43 (2.22–5.29), <0.001	3.41 (1.31–8.88), 0.012	5.79 (2.45–13.71), <0.001	1.24 (1.06–1.43), 0.005	1.68 (1.19–2.18), <0.001
Yes					
No (reference)					

^1^ Multiple logistic regression analysis, ^2^ multiple Poisson regression analysis, ^3^ multiple linear regression analysis. Abbreviations: CI—confidence interval; CIS—carcinoma in situ; IRR—incidence rate ratio; OR—odds ratio.

**Table 3:** Comparison of bladder cancer features between patients with gross vs. microscopic hematuria.

Variable	Type of Hematuria		*p*-Value
	Gross	Microscopic	
Bladder cancer grade, n (%)			
PUNLMP/low grade	143 (42.1)	29 (58)	0.034 ^1^
High-grade	197 (57.9)	21 (42)	
Concomitant CIS, n (%)	32 (9.4)	1 (2)	0.079 ^1^
Bladder cancer stage, n (%)			
Ta	165 (48.5)	37 (74)	
T1	107 (31.5)	11 (22)	0.002 ^1^
T2	68 (20)	2 (4)	
Morphological characteristics, median (IQR)			
Number of tumors	1 (1–3)	1 (1–2)	0.369 ^2^
Total tumor size (cm)	3.5 (2.35–5)	2 (1.2–3.4)	<0.001 ^2^

^1^ Chi-square test; ^2^ Mann–Whitney test. Abbreviations: CIS—carcinoma in situ; IQR—interquartile range; PUNPLMP—papillary urothelial neoplasm of low malignant potential.

**Table 4:** Results of multiple regression analyses examining the impact of patient demographics and type of hematuria on the histopathological grade, stage, number, and size of bladder cancer in patients with hematuria.

	Dependent Variables				
Independent Variables	High-Grade vs. Non-High-Grade	Concomitant CIS vs. no CIS	T2 Stage vs. Ta/T1 Stage	Number of Tumors	Tumor Size
	OR (95% CI), *p*-Value ^1^	OR (95% CI), *p*-Value ^1^	OR (95% CI), *p*-Value ^1^	IRR (95% CI), *p*-Value ^2^	B coefficient (95% CI), *p*-Value ^3^
Sex	1.65 (1.05–2.59), 0.032	2.47 (0.92–6.63), 0.072	0.76 (0.44–1.34), 0.349	1.02 (0.88–1.19), 0.140	−0.2 (−0.8–0.39), 0.508
Male					
Female (reference)					
Age	1.04 (1.01–1.06), 0.003	0.99 (0.96–1.04) 0.892	1.01 (0.98–1.04), 0.423	1.02 (1.01–1.024), <0.001	0.06 (0.03–0.09), <0.001
Smoking history	0.93 (0.59–1.45), 0.733	0.91 (0.41–1.99), 0.814	0.86 (0.49–1.52), 0.605	0.94 (0.81–1.09), 0.438	−0.26 (−0.84–0.32), 0.381
Yes					
No (reference)					
Number of comorbidities	0.88 (0.76–1.03), 0.107	0.82 (0.61–1.09), 0.821	0.92 (0.75–1.12), 0.404	0.97 (0.92–1.02), 0.241	−0.165 (−0.36–0.03), 0.102
Type of hematuria	2.07 (1.12–3.84), 0.020	5.28 (0.7–39.72), 0.106	6.03 (1.42–25.49), 0.015	1.16 (0.94–1.44), 0.191	1.8 (0.99–2.6), <0.001
Gross					
Microscopic (reference)					

^1^ Multiple logistic regression analysis, ^2^ multiple Poisson regression analysis, ^3^ multiple linear regression analysis. Abbreviations: CI—confidence interval; CIS—carcinoma in situ; IRR—incidence rate ratio; OR—odds ratio.

## Data Availability

The authors confirm that all original data presented in this study are available and can be obtained upon request from the corresponding author.

## Supplementary Materials

The following supporting information can be downloaded at: https://www.mdpi.com/article/10.3390/jcm12134259/s1, Figure S1: Examples of histopathological microscopic images of bladder cancer, classified according to the World Health Organization and the TNM Classification for Bladder Cancer: (a) low-grade, Ta stage; (b) carcinoma in situ (CIS); (c) high-grade, T1 stage; (d) high-grade, T2 stage. The tissue samples were stained with hematoxylin-eosin, and corresponding images were captured at a magnification of 200× (a,c,d) and a magnification of 100× (b). Figure S2: Examples of intraoperative images of bladder cancer and its morphological features: (a) resection loop of the resectoscope, measuring 6 mm (HF-resection electrode, loop, 24 Fr., Olympus Winter and IBE GMBH); (b) tumor with a largest dimension of 4 mm; (c) tumor with a largest dimension of 8 mm; (d) tumor with a largest dimension of 10 mm.

## References

[1] Sung H., Ferlay J., Siegel R.L., Laversanne M., Soerjomataram I., Jemal A., Bray F. (2021). Global Cancer Statistics 2020: GLOBOCAN Estimates of Incidence and Mortality Worldwide for 36 Cancers in 185 Countries. CA Cancer J. Clin..

[2] Miyazaki J., Nishiyama H. (2017). Epidemiology of Urothelial Carcinoma. Int. J. Urol. Off. J. Jpn. Urol. Assoc..

[3] Mariani A.J., Mariani M.C., Macchioni C., Stams U.K., Hariharan A., Moriera A. (1989). The Significance of Adult Hematuria: 1000 Hematuria Evaluations Including a Risk-Benefit and Cost-Effectiveness Analysis. J. Urol..

[4] Arianayagam R., Arianayagam M., Rashid P. (2011). Bladder Cancer--Current Management. Aust. Fam. Physician.

[5] Kirkali Z., Chan T., Manoharan M., Algaba F., Busch C., Cheng L., Kiemeney L., Kriegmair M., Montironi R., Murphy W.M. (2005). Bladder Cancer: Epidemiology, Staging and Grading, and Diagnosis. Urology.

[6] Tohi Y., Miyauchi Y., Yamasaki M., Fujiwara K., Harada S., Matsuda I., Ito A., Matsuoka Y., Kato T., Taoka R. (2022). Incidental Bladder Cancer Found on Cystoscopy during Prostate Biopsy: Prevalence, Pathological Findings, and Oncological Outcome. Urol. Int..

[7] Rayn K.N., Hale G.R., Bloom J.B., Gold S.A., Carvalho F.L.F., Mehralivand S., Czarniecki M., Wood B.J., Merino M.J., Choyke P. (2018). Incidental Bladder Cancers Found on Multiparametric MRI of the Prostate Gland: A Single Center Experience. Diagn. Interv. Radiol. Ank. Turk..

[8] Ramirez D., Gupta A., Canter D., Harrow B., Dobbs R.W., Kucherov V., Mueller E., Streeper N., Uhlman M.A., Svatek R.S. (2016). Microscopic Haematuria at Time of Diagnosis Is Associated with Lower Disease Stage in Patients with Newly Diagnosed Bladder Cancer. BJU Int..

[9] Antoni S., Ferlay J., Soerjomataram I., Znaor A., Jemal A., Bray F. (2017). Bladder Cancer Incidence and Mortality: A Global Overview and Recent Trends. Eur. Urol..

[10] Siow W.Y., Yip S.K., Ng L.G., Tan P.H., Cheng W.S., Foo K.T. (2000). Renal Cell Carcinoma: Incidental Detection and Pathological Staging. J. R. Coll. Surg. Edinb..

[11] Barocas D.A., Boorjian S.A., Alvarez R.D., Downs T.M., Gross C.P., Hamilton B.D., Kobashi K.C., Lipman R.R., Lotan Y., Ng C.K. (2020). Microhematuria: AUA/SUFU Guideline. J. Urol..

[12] Humphrey P.A., Moch H., Cubilla A.L., Ulbright T.M., Reuter V.E. (2016). The 2016 WHO Classification of Tumours of the Urinary System and Male Genital Organs-Part B: Prostate and Bladder Tumours. Eur. Urol..

[13] Amin M.B., Edge S., Greene F., Byrd D.R., Brookland R.K., Washington M.K., Gershenwald J.E., Compton C.C., Hess K.R., Sullivan D.C. (2017). AJCC Cancer Staging Manual.

[14] Gaya J.M., Territo A., Woldu S., Schwartzmann I., Verri P., González-Pérez L., Cózar J.M., Miñana B., Medina R.A., de la Rosa-Kehrmann F. (2022). Incidental Diagnosis of Bladder Cancer in a National Observational Study in Spain. Actas Urol. Esp..

[15] Kamecki H., Dębowska M., Nyk Ł., Przewor A., Demkow T., Sosnowski R. (2022). The Clinical Features of Incidentally Diagnosed Urothelial Bladder Cancer: A Retrospective Data Analysis. Urol. Int..

[16] Nieder A.M., Lotan Y., Nuss G.R., Langston J.P., Vyas S., Manoharan M., Soloway M.S. (2010). Are Patients with Hematuria Appropriately Referred to Urology? A Multi-Institutional Questionnaire Based Survey. Urol. Oncol..

[17] Wakai K., Utsumi T., Oka R., Endo T., Yano M., Kamijima S., Kamiya N., Hiruta N., Suzuki H. (2016). Clinical Predictors for High-Grade Bladder Cancer before First-Time Transurethral Resection of the Bladder Tumor: A Retrospective Cohort Study. Jpn. J. Clin. Oncol..

[18] Shapur N., Pode D., Katz R., Shapiro A., Yutkin V., Pizov G., Appelbaum L., Zorn K.C., Duvdevani M., Landau E.H. (2011). Predicting the Risk of High-Grade Bladder Cancer Using Noninvasive Data. Urol. Int..

[19] Warrick J.I., Knowles M.A., Yves A., van der Kwast T., Grignon D.J., Kristiansen G., Egevad L., Hartmann A., Cheng L. (2020). Report from the International Society of Urological Pathology (ISUP) Consultation Conference on Molecular Pathology of Urogenital Cancers. II. Molecular Pathology of Bladder Cancer: Progress and Challenges. Am. J. Surg. Pathol..

[20] Lin M.G., Hong Y.K., Zhang Y., Lin B.B., He X.J. (2018). Mechanism of LncRNA DUXAP8 in Promoting Proliferation of Bladder Cancer Cells by Regulating PTEN. Eur. Rev. Med. Pharmacol. Sci..

[21] Sylvester R.J., van der Meijden A.P.M., Oosterlinck W., Witjes J.A., Bouffioux C., Denis L., Newling D.W.W., Kurth K. (2006). Predicting Recurrence and Progression in Individual Patients with Stage Ta T1 Bladder Cancer Using EORTC Risk Tables: A Combined Analysis of 2596 Patients from Seven EORTC Trials. Eur. Urol..

[22] Liu S., Hou J., Zhang H., Wu Y., Hu M., Zhang L., Xu J., Na R., Jiang H., Ding Q. (2015). The Evaluation of the Risk Factors for Non-Muscle Invasive Bladder Cancer (NMIBC) Recurrence after Transurethral Resection (TURBt) in Chinese Population. PLoS ONE.

[23] Sylvester R.J., Rodríguez O., Hernández V., Turturica D., Bauerová L., Bruins H.M., Bründl J., van der Kwast T.H., Brisuda A., Rubio-Briones J. (2021). European Association of Urology (EAU) Prognostic Factor Risk Groups for Non-Muscle-Invasive Bladder Cancer (NMIBC) Incorporating the WHO 2004/2016 and WHO 1973 Classification Systems for Grade: An Update from the EAU NMIBC Guidelines Panel. Eur. Urol..

[24] Tian Z., Meng L., Wang X., Diao T., Hu M., Wang M., Zhang Y., Liu M. (2021). Predictive Nomogram and Risk Factors for Lymph Node Metastasis in Bladder Cancer. Front. Oncol..

[25] Tang F., He Z., Lu Z., Wu W., Chen Y., Wei G., Liu Y. (2019). Application of Nomograms in the Prediction of Overall Survival and Cancer-Specific Survival in Patients with T1 High-Grade Bladder Cancer. Exp. Ther. Med..

[26] Wang J., Wu Y., He W., Yang B., Gou X. (2020). Nomogram for Predicting Overall Survival of Patients with Bladder Cancer: A Population-Based Study. Int. J. Biol. Markers.

[27] Chen D., Luo Z., Ye C., Luo Q., Fan W., Chen C., Liu G. (2022). Constructing and Validating Nomograms to Predict Risk and Prognostic Factors of Distant Metastasis in Urothelial Bladder Cancer Patients: A Population-Based Retrospective Study. BMC Urol..

[28] Carrion R., Seigne J. (2002). Surgical Management of Bladder Carcinoma. Cancer Control J. Moffitt Cancer Cent..

[29] Kamecki H., Dębowska M., Poleszczuk J., Demkow T., Przewor A., Nyk Ł., Sosnowski R. (2023). Incidental Diagnosis of Urothelial Bladder Cancer: Associations with Overall Survival. Cancers.

[30] Wurcel V., Cicchetti A., Garrison L., Kip M.M.A., Koffijberg H., Kolbe A., Leeflang M.M.G., Merlin T., Mestre-Ferrandiz J., Oortwijn W. (2019). The Value of Diagnostic Information in Personalised Healthcare: A Comprehensive Concept to Facilitate Bringing This Technology into Healthcare Systems. Public Health Genom..

[31] Zhang J., Gerst S., Lefkowitz R.A., Bach A. (2007). Imaging of Bladder Cancer. Radiol. Clin. N. Am..

[32] Gontero P., Compérat E., Dominguez Escrig J.L., Liedberg F., Mariappan P., Masson-Lecomte A., Mostafid A.H., van Rhijn B.W.G., Rouprêt M., Seisen T. (2023). EAU Guidelines on Non-Muscle-Invasive Bladder Cancer (TaT1 and CIS).

[33] Krabbe L.M., Svatek R.S., Shariat S.F., Messing E., Lotan Y. (2015). Bladder Cancer Risk: Use of the PLCO and NLST to Identify a Suitable Screening Cohort. Urol. Oncol..

