# Supplementary figures and images for: The Impact of the Initial Clinical Presentation of Bladder Cancer on Histopathological and Morphological Tumor Characteristics
Source: J Clin Med. 2023 Jun 25;12(13):4259. doi: 10.3390/jcm12134259 (PMC10342402; doi:10.3390/jcm12134259)

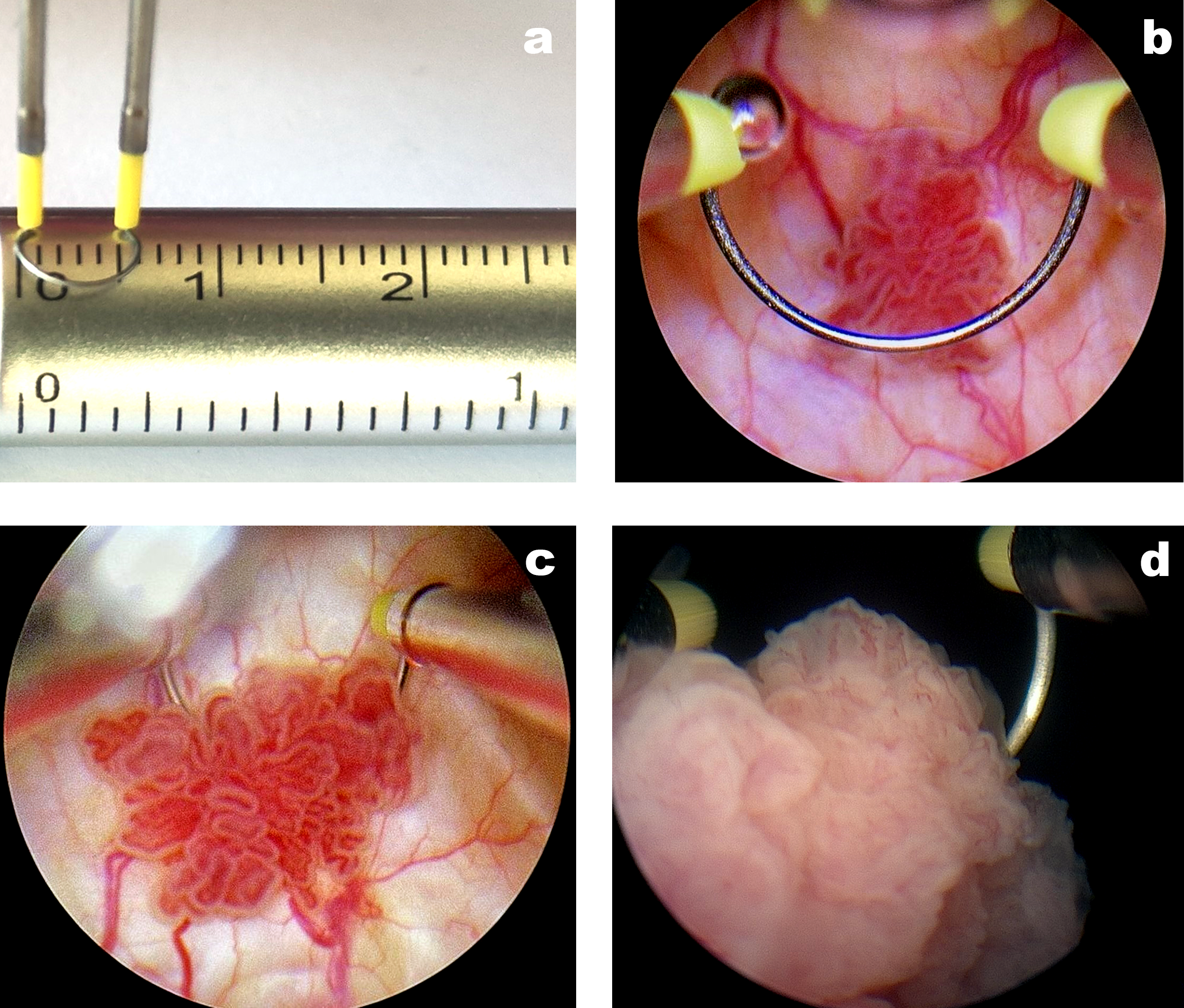

Supplement: Supplementary file 1 [file jcm-12-04259-s001.zip › Figure S2.tif]
